# Major problems, current characteristics and future career plans of obstetrics and gynecology residents in Turkey

**DOI:** 10.4274/tjod.galenos.2019.75428

**Published:** 2019-10-10

**Authors:** Selçuk Erkılınç, Murat Yassa, Buğra Coşkun, Onur İnce, Ateş Karateke

**Affiliations:** 1 Isparta City Hospital, Clinic of Gynecologic Oncology, Isparta, Turkey; 2 Bartın State Hospital, Clinic of Obstetrics and Gynecology, Bartın, Turkey; 3 Liv Hospital, Clinic of Obstetrics and Gynecology, Ankara, Turkey; 4 Kütahya University of Health Sciences, Faculty of Medicine, Department of Obstetrics and Gynecology, Kütahya, Turkey; 5 İstanbul Medeniyet University Faculty of Medicine, Department of Obstetrics and Gynecology, İstanbul, Turkey

**Keywords:** Obstetrics and gynecology department, hospital, residency, training

## Abstract

**Objective:**

To evaluate the current problems and future career plans of obstetrics and gynecology residents in Turkey.

**Materials and Methods:**

In this cross-sectional study, a survey was conducted with 143 trainees from 25 cities in different regions of Turkey. The questionnaire, which was sent via e-mail to all available trainees, consisted of four parts: information on hospitals, number and variety of surgical interventions, scientific activities, and current problems. Descriptive statistics were used to analyze participants’ responses.

**Results:**

The mean number of trainees in each hospital was 24 in education and research hospitals and 15 in university hospitals (p<0.001). Perinatology, oncology, and infertility clinics were present in about 70% of the hospitals, and there was no difference in this regard between public and university hospitals. Most trainees (68.5%) complained about being alone in an outpatient clinic. Third-year trainees from training and research hospitals performed a significantly higher number of vaginal births than those at universities (p=0.035). Most trainees complained about their workload during their residency in both training and research hospitals (74.4%) and university hospitals (66%). The three most common plans for the future were to attend a subspecialty program in the field of obstetrics and gynecology (28%), to pursue an academic career (23.1%), and to work in a private hospital (21%).

**Conclusion:**

Extremely long work hours, excessive workload, many monthly duties, and lack of supervision at outpatient clinics were found to be the major problems of the obstetrics and gynecology residents in Turkey. The most common future plan of the residents was to attend a subspecialty program in the field of obstetrics and gynecology.

**PRECIS:** In this study, the current problems and future expectations of obstetrics and gynecology residents across the nation were evaluated.

## Introduction

Turkish Trainees of Obstetrics and Gynecology is an organization that has collaborated with the European Board College for Obstetrics and Gynecology and European Network Trainees of Obstetrics and Gynecology (ENTOG) since 2010^(1)^. This collaboration facilitated a mutual understanding of educational models across Europe. There are advantages and disadvantages of training in obstetrics and gynecology (OBGYN) specialism in Turkey. The population of Turkey is about 80 million^(2)^ and the annual number of trainees in OBGYN is about 1200. The low trainee to population ratio results in long working hours and fatigue. On the other hand, the upside of this low ratio is the high number of operations per trainee^(3)^. Training in OBGYN takes place in two types of hospitals in Turkey. Training and research hospitals are public hospitals that are affiliated to the ministry of health and render most of the healthcare service to the general population^(4)^. University hospitals are self-governing hospitals that deal with more complex cases and their work load is lower than public hospitals. Despite some technical limitations, these two types of hospitals have nationwide coverage, even in rural areas. There are no operational national mobilization and external rotation programs, so trainees have to complete all of their rotations in the same hospital. To the best of our knowledge, there is no study evaluating current problems and characteristics of OBGYN training in Turkey. This survey evaluates the current conditions and issues regarding OBGYN residency. We believe that identifying the issues is the first step to developing the relevant solutions.

## Materials and Methods

The study was conducted in cross-sectional design. The OBGYN training institutions in Turkey were determined relying on the information from Measuring, Selection and Placement Center’s web page^(5)^. Fifty-six institutions were identified. The names of trainees were collected from the webpages of the 44 of the institutions that provided this information. The contact information of the trainees was collected through social networks (Facebook/tjodasistan). A survey was sent via e-mail to 568 trainees whose contact information was available. One hundred forty-three of the 568 trainees (25.3%) responded to the survey. These 143 trainees were from 25 cities from all the different regions of Turkey. Informed consent was obtained from these trainees. The survey was an online multiple choice questionnaire. The questionnaire consisted of four parts, concerning information on hospitals, number and variety of surgical interventions, scientific activities and problems. More specifically, the questions asked for the city, year of training, presence of departments of gynecologic oncology, perinatology, infertility, whether the hospital was public or university, the number of trainees at the hospital, number of deliveries per day, number of night shifts, supervision at outpatient clinics and operations, time of first deliveries and cesarean section, number of operations in gynecology, perinatology, infertility and oncology, utility of rotations to other specialties, number of scientific meetings, and number of international meetings. The questions on the problems of trainees included day-after duties, workload, working hours, mobbing, lack of support for scientific activities, lack of supervision in outpatient clinics, vacations, satisfaction with salary, and future plans. The data were collected using Google documents and analyzed with the IBM SPSS statistics ver. 21.0 (IBM Corp., Armonk, NY) software package. Continuous data are presented as mean ± standard deviation or median (minimum-maximum), and categorical data are reported as number and percentage. Continuous data were compared using the Independent Sample t-test or Mann-Whitney U tests. Categorical data were compared using the chi-square test.

## Results

**Figure 1:**
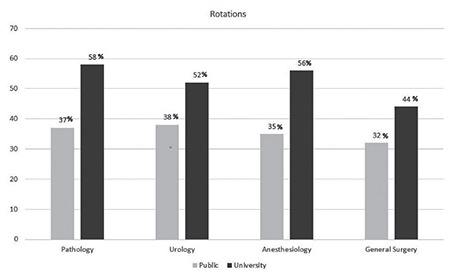
Percentage of trainees scoring rotations as inefficient

**Figure 2:**
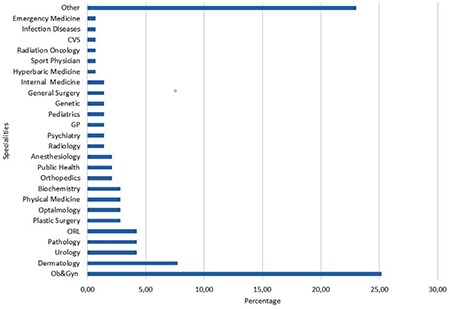
What would you choose if you had a chance to change your specialty? Ob&Gyn: Obstetrics gynecology, ORL: Otorhinolaryngology, CVS: Cardiovascular surgery

**Figure 3:**
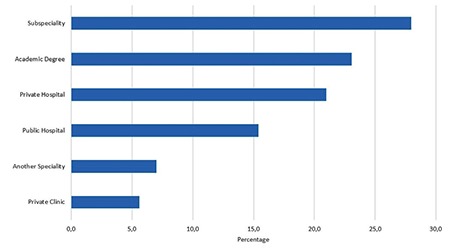
Future plans of residents in Turkey

One hundred forty-three trainees from 56 institutions, 27 university hospitals and 29 education and research hospitals (a type of public hospital that provides training to residents), were included in the study. The percentage of trainees with 1, 2, 3, and 4 years of training were 16%, 35%, 37%, and 55%, respectively. The mean number of trainees in training and research hospitals and university hospitals was 24 and 15 respectively. Although the median number of births per day was higher in public hospitals than in university hospitals, there was no significant difference between university and public hospitals (20 vs. 10; p>0.05). Perinatology, oncology and infertility clinics were present in about 70% of the hospitals and the difference between public and university hospitals was not significant. Only about 7% of the trainees could do post-call off. The trainees preferred to be a trainee in OBGYN as a first choice with a rate of 61%. Most of the trainees complained about being alone in outpatient clinics, with 68.5% reporting no supervision. The first labor during training was performed in the first month by 70% of the trainees. The comparison of the data between public and university hospitals is shown in Table 1. The number of operations performed by the trainees under supervision is shown in Table 2. All operation rates were found to be similar between the two types of hospitals except for vaginal births for 3^rd^ year trainees. Trainees at public hospitals in the 3^rd^ year performed a significantly higher number of vaginal births than those at university hospitals. About half of the trainees assessed the utility of rotations with the lowest score. The trainees ranked pathology as the branch with the least utility during training. The utility rates of the other rotations from the point of view of the trainees are given in Figure 1. The trainees in training and research hospitals and university hospitals complained about the workload at rates of 74.4% and 66%, respectively. Excessive numbers of nightshifts bothered 46% and 39% of trainees in public hospitals and university hospitals, respectively. Trainees also identified mobbing as a problem at 30% and 22% in public and university hospitals, respectively. Working alone in an outpatient clinic without a supervisor was identified as a problem by 61% and 58.5% of the trainees in public and university hospitals. Problems related to work and their review by the trainees are given in Table 3. If given the chance to choose their specialty again, OBGYN remained the most popular choice of the trainee at 25.2%. Dermatology, urology, and pathology specialties were chosen by 7.2%, 4.2%, and 4.2% of the trainees, respectively. The least popular specialties were cardiovascular surgery, infectious diseases, and emergency medicine (0.7% for each) as demonstrated in Figure 2. After completing the residency, the three most common preferences for the future career plans were to attend a subspecialty fellowship program in the field of OBGYN, to pursue an academic career, and to work in a private hospital (28%, 23.1%, and 21%, respectively). Seven percent of the trainees, on the other hand, planned to pursue a career in another specialty. With regard to future places of work, 15.4% of the residents were planning to work in a public hospital, and 5.6% were planning to have their own private clinic, as demonstrated in Figure 3.

## Discussion

This study highlights the main problems of OBGYN residents in Turkey by providing objective baseline data that can be compared with other European countries. The evidence points to an excessive workload on OBGYN residents with the benefit of an early start to vaginal/cesarean births and performing a large number of gynecologic surgeries. Half of the (54.6%) trainees planned to work in the private sector, and 7% planned to switch to another specialty. The number of residents in OBGYN training programs is inadequate to meet demands of the growing adult female population, and the current shortage of OBGYNN residents is projected to worsen in the future^(6)^. Residents were found to experience significantly more burnout, have a higher risk for psychological morbidity, and lower career satisfaction rates relative to attending surgeons^(7)^. Becker et al.^(8)^ assessed burnout and depression rates in 118 residents from 23 different randomly selected OBGYN residency programs in the United States. Almost 90% of the residents were found to have moderate burnout and one-third showed signs of depression. Working hours are known to be a powerful predictor of burnout, psychiatric morbidity, and decreased work-life balance^(7)^. Alston et al.^(9)^ interviewed 226 medical students who stood up for an OBGYN residency position. The greatest concern about OBGYN was the long work hours for 65% of the students. The current study revealed that 59.4% of the residents had night-shifts every other day in their first half of the residency. Only 5.6% of the residents had the chance to return home after the in-hospital call. One-third of the residents claimed that they had no chance for a yearly vacation. An observational, descriptive, and cross-sectional study conducted in 25 different ENTOG member countries found the average number of weekly working hours for 6056 OBGYN trainees as 51.6 hours, with an average night-shift number of 5 (range, 2-9) per month^(10)^. The burnout effects, physiologic morbidities, and potential safety concerns caused by these extreme working hours are still unknown and need to be investigated. Impaired surgeon performance due to sleep deficiency has been associated with serious medical errors. However, it was hypothesized that home call design may compromise resident clinical experience and satisfaction, although it has allowed for compliance with duty hour requirements^(11)^. We believe that extending the duration of OBGYN residency may be better for training and work-life balance than transforming in-hospital calls into house calls. In this vein, ENTOG has officially suggested that the minimum duration of training should be at least five years to improve harmonization^(10)^. In an interview study, it was found that the most important factor in selecting an OBGYN residency among medical students was the overall surgical training, particularly training in laparoscopic surgery^(12)^. Cadish and Muffly^(13)^ conducted a retrospective cohort study of recent OBGYN residency graduates to estimate the average simple hysterectomy volume performed in the United States. It was found that recent graduates performed an average of 4 hysterectomies annually in their first five years after residency. The most common route was the abdominal route with 41.6%, and the second common route was laparoscopy with 32.4%. In the current study, a trainee primarily performed an average of 50 abdominal hysterectomies, 5 laparoscopic hysterectomies, and 10 vaginal hysterectomies in the last year before graduation. The number of performed surgeries during the residency is the main upside of the excessive work hours and night shifts. A recent study conducted in six different university in the United States revealed that 43% of residents were planning to enter private practice and only 19.4% of those were planning to pursue an academic career^(14)^. In the current study, comparable findings were observed with 44.6% of the residents planning to work in the private sector and 23.1% planning an academic career. The obstacles in the way of pursuing an academic career should be investigated further. In the present study, medicolegal issues were found to worry 31% and 22.6% of the residents in public and university hospitals, respectively. Current data suggest that medico-legal training during OBGYN residency is inadequate^(15)^. A large survey study showed that more than 20% of the fourth-year OBGYN residents had already been in a lawsuit^(16)^ and more than half of those indicated that they had not received adequate training on legal matters. In a prospective survey study, 35% of residents stated that they had been planning for a fellowship program only because of malpractice concerns. In Turkey, all procedures are under a specialist’s responsibility. However, malpractice insurance is still a crucial support for the residents. In Turkey, malpractice insurances are semi-subsidized, and should be encouraged more. This study is the first to objectively assess the problems and career plans of OBGYN residents in Turkey. Its major limitations are the lack of subgroup analysis for sex distribution and the low response rate. At the same time, the respondents were from 25 cities from all the different regions of Turkey, which bodes well for the representativeness of the sample.

Based on the analysis of the survey results, one-to-one interviews and concilium with the ENTOG, the following suggestions were identified in order to improve the job satisfaction, quality of training, burnout and depression levels, and liability and medicolegal preparedness:

- Minimum duration of OBGYN residency should be five years to improve harmonization with Europe and reduce long working hours,

- Post-call leaves should be well balanced,

- Supervision in outpatient clinics should be provided,

- Generalized national qualification exams should be compatible with ENTOG member countries,

- A standardized national logbook should be formed to standardize training,

- Rotations should be functional, and external rotations should be implemented in university hospitals.

## Conclusion

OBGYN has been the top preferred residency position in Turkey in the past. OBGYN trainees’ career expectations and future plans appear to be changing. Extremely long work hours, excessive workload, high number of duties per month, and no supervision in outpatient clinics were found to be the major issues. Identifying these issues is critical to improve the quality of training and demand/supply balance for residency.

## Figures and Tables

**Table 1:**
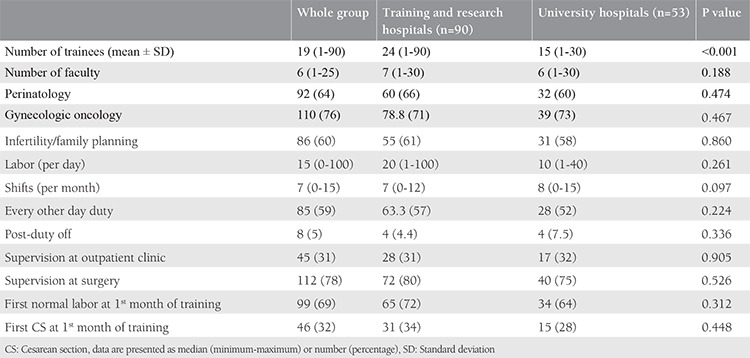
Demographic data of hospitals and residents in obstetrics and gynecology in Turkey

**Table 2:**
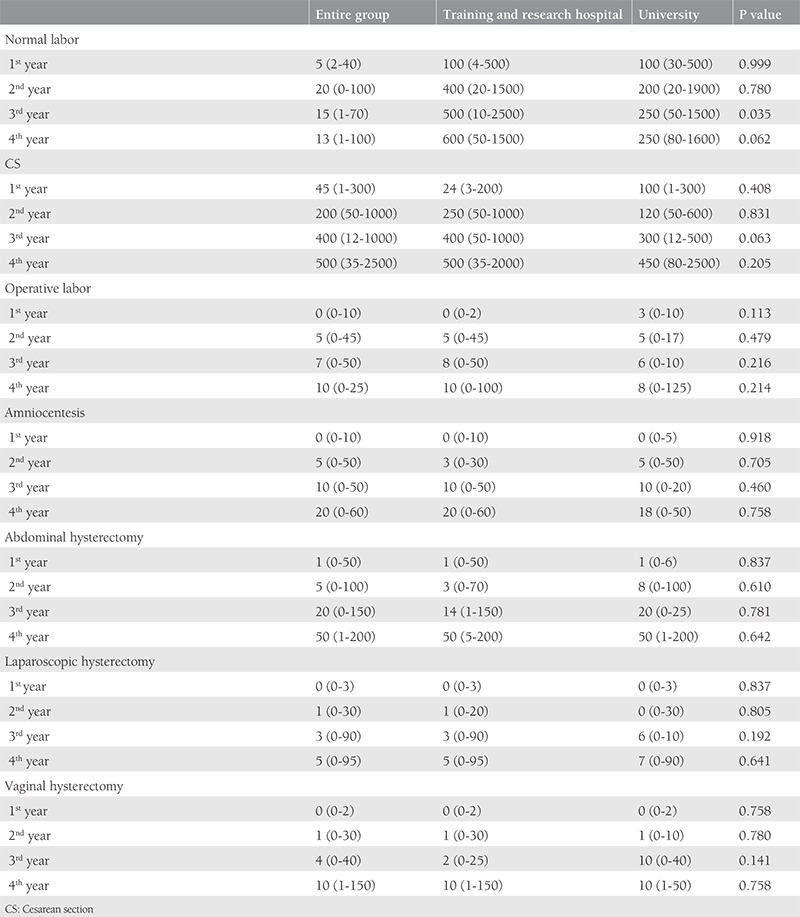
Number of operations performed by trainees under supervision

**Table 3:**
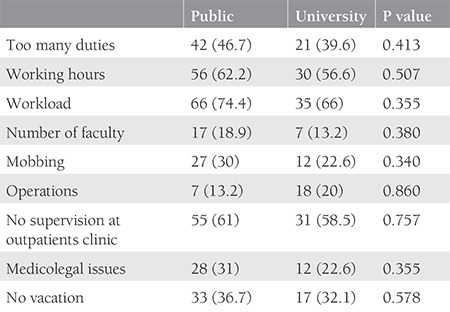
Percentage of trainees who scored problems as most annoying
